# α-Synuclein pathology in post-mortem retina and optic nerve is specific for α-synucleinopathies

**DOI:** 10.1038/s41531-023-00570-5

**Published:** 2023-08-28

**Authors:** Frederique J. Hart de Ruyter, Tjado H. J. Morrema, Jurre den Haan, Gina Gase, Jos W. R. Twisk, Johannes F. de Boer, Philip Scheltens, Femke H. Bouwman, Frank D. Verbraak, Annemieke J. M. Rozemuller, Jeroen J. M. Hoozemans

**Affiliations:** 1 Department of Pathology, Amsterdam UMC location Vrije Universiteit Amsterdam, De Boelelaan 1117, Amsterdam, The Netherlands; 2 Department of Neurology and Alzheimer Center Amsterdam, Amsterdam UMC location Vrije Universiteit Amsterdam, De Boelelaan 1117, Amsterdam, The Netherlands; 3 Department of Epidemiology and Data Science, Vrije Universiteit Amsterdam, De Boelelaan 1117, Amsterdam, The Netherlands; 4 Department of Physics and Astronomy and LaserLaB, Vrije Universiteit Amsterdam, De Boelelaan 1081, Amsterdam, The Netherlands; 5 Department of Ophthalmology, Amsterdam UMC location Vrije Universiteit Amsterdam, De Boelelaan 1117, Amsterdam, The Netherlands; 6 Amsterdam Neuroscience, Neurodegeneration, Amsterdam, The Netherlands

**Keywords:** Parkinson's disease, Visual system, Dementia

## Abstract

There is increasing interest in studying retinal biomarkers for various neurodegenerative diseases. Specific protein aggregates associated with neurodegenerative diseases are present in the retina and could be visualised in a non-invasive way. This study aims to assess the specificity and sensitivity of retinal α-synuclein aggregates in neuropathologically characterised α-synucleinopathies, other neurodegenerative diseases and non-neurological controls. Post-mortem eyes (*N* = 99) were collected prospectively through the Netherlands Brain Bank from donors with Parkinson’s disease (and dementia), dementia with Lewy bodies, multiple system atrophy, Alzheimer’s disease, other neurodegenerative diseases and non-neurological controls. Multiple retinal and optic nerve cross-sections were immunostained with anti-α-synuclein antibodies (LB509, KM51, and anti-pSer129) and assessed for aggregates and inclusions. α-Synuclein was observed as Lewy neurites in the retina and oligodendroglial cytoplasmic inclusions in the optic nerve and was highly associated with Lewy body disease (*P* < 0.001) and multiple system atrophy (*P* = 0.001). In all multiple system atrophy cases, the optic nerve showed oligodendroglial cytoplasmic inclusions, while retinal Lewy neurites were absent, despite coincidental brain Lewy pathology. With high specificity (97%) and sensitivity (82%), retinal/optic nerve α-synuclein differentiates primary α-synucleinopathies from other cases and controls. α-Synuclein pathology occurs specifically in the retina and optic nerve of primary α-synucleinopathies as opposed to other neurodegenerative diseases—with and without α-synuclein co-pathology—and controls. The absence of retinal Lewy neurites in multiple system atrophy could contribute to the development of an in vivo retinal biomarker that discriminates between Lewy body disease and multiple system atrophy.

## Introduction

The retina and optic nerve are embryonically derived from the central nervous system and show similar structural and functional characteristics to the brain^1^. Therefore, the retina is considered a potential source of biomarkers for neurodegenerative brain diseases. The optic nerve consists of glial cells and the axons of retinal ganglion cells, which extend as the optic tract to the lateral geniculate nucleus and superior colliculus, forming a direct connection with the brain. The retina contains ganglion cells displaying similarities with neurons of the central nervous system, interneurons, and different types of glial cells^2^. Existing data suggests that the retina mirrors ongoing pathology associated with neurodegenerative diseases in the brain^3–10^, supporting the potential of in-vivo retinal imaging as a non-invasive diagnostic approach.

Several neurodegenerative diseases are characterised by the aggregation of α-synuclein (αSyn) and therefore termed α-synucleinopathies^11^. α-Synucleinopathies—of which Parkinson’s disease is the most common form—can be subdivided into two major classes: Lewy body disease and multiple system atrophy^12,13^. Lewy body disease encompasses the clinically defined entities Parkinson’s disease, Parkinson’s disease dementia and dementia with Lewy bodies. It is neuropathologically characterised by neuronal αSyn aggregation forming Lewy bodies and Lewy neurites^11^. Multiple system atrophy is characterised by oligodendroglial cytoplasmic inclusions (GCIs). These neuropathological aggregates are associated with neuronal death and demyelination^14^. Pathological formation of αSyn aggregates are thought to be caused by the binding of cytoplasmic αSyn with lipid membranes disrupting their role in regulating neurotransmission and synaptic function^15,16^. Lewy bodies and GCIs are primarily composed of αSyn phosphorylated at serine 129 (pSer129), which is why this phosphorylated αSyn epitope is considered a marker for pathology and suggested as a potential diagnostic and progression biomarker in Parkinson’s disease^17,18^. Despite their shared presence of αSyn aggregates, α-synucleinopathies show significant clinical and pathological heterogeneity.

Under normal conditions, αSyn is endogenously present in all retinal layers in cell bodies and processes^19,20^. In Parkinson’s disease, native αSyn was found as neuronal, globular inclusions in the inner nuclear layer and ganglion cell layer^8^. Since αSyn pSer129 has gained interest as a key marker for pathology, different studies on the retina have reported the presence of αSyn pSer129 specifically in Parkinson’s disease^7,9^ and dementia with Lewy bodies^7^ as opposed to control cases. Ortuño-Lizarán and colleagues^9^_,_ studying whole-mount retinas of Parkinson’s disease cases, reported αSyn pSer129 deposits resembling brain Lewy bodies. αSyn pSer129 was observed in ganglion cell perikarya, axonal fibres and dendrites. Additional αSyn pSer129 immunostaining on cross-sections showed inclusions in the inner nuclear and ganglion cell layers resembling characteristic Lewy bodies and neurites. Despite these significant findings, one study could not confirm the presence of αSyn pathology in the retinas of Parkinson’s disease as opposed to controls^21^.

It has been reported that the level of αSyn pathology in the retina correlated positively with the severity of Parkinson’s disease and may therefore serve as a biomarker for the disease^9^. In-vivo examination of αSyn in the retina may provide insight into the pathogenesis of α-synucleinopathies and the potential efficacy of therapeutic interventions. While previous studies have already established the presence of Lewy pathology in the retina in Parkinson’s disease^7,9^, dementia with Lewy bodies^7^ and incidental Lewy body disease^9^, the ophthalmologic occurrence of αSyn in multiple system atrophy has not been reported before. Moreover, it remains unclear whether αSyn is present in the retina of cases with other neurodegenerative diseases and with αSyn co-pathology in the brain, for instance, in the presence of incidental, amygdala-only αSyn pathology^22^. This study evaluated the presence of ophthalmologic αSyn pathology in a large cohort with different neurodegenerative diseases, including primary α-synucleinopathies and other neurodegenerative disorders with and without αSyn co-pathology, and non-neurological controls.

## Results

### Cohort description

Individual case details are reported in Supplementary Tables 1 and 2, and group demographics based on neuropathological diagnosis are described in Table 1. No significant group difference was found for age at death (*P* = 0.16), sex (*P* = 0.08) or post-mortem interval (*P* = 0.06). Parkinson’s disease (dementia) had a significantly higher disease duration than all other groups (*P* < 0.001). A significant difference was found between groups for visual hallucinations and dementia (*P* < 0.001). α-Synucleinopathies had a significantly higher Braak LB stage (*P* < 0.001) and LPC stage (*P* < 0.001).

**Table 1:** Group demographics of cases used in this study based on clinicopathological diagnosis.

	Controls	Parkinson’s disease (dementia)	Dementia with Lewy bodies	Multiple system atrophy	Alzheimer’s disease	Other neurodegenerative diseases
				MSA-p, MSA-c/p		Primary tauopathies, FTLD, ALS, MS, Fragile X syndrome, vascular dementia, hydrocephalus
	*n* = 25	*n* = 21	*n* = 5	*n* = 7	*n* = 19	*n* = 22
Female, *n*	18	7	3	5	12	9
Age at death mean ± SD	72 ± 16	77 ± 9	81 ± 8	70 ± 9	77 ± 9	70 ± 14
Disease duration median; min–max	n/a	192 (37–324)	72 (48–84)	60 (24–120)	108 (12–312)	84 (24–180)
Presence of visual hallucinations, *n*	0	13	5	0	1	2
Presence of dementia, *n*	0	9	3	1	16	9
Braak stage for LB median; min–max	0 (0–4)	6 (3–6)	6 (4–6)	4 (3–5)	0 (0–4)	0 (0–3)
LPC stage	0 (0–4)	5 (3–5)	5 (5)	5 (3–5)	0 (0–4)	0 (0–3)
Post-mortem interval eyes median; min–max	8 (3–30)	6 (4–10)	7 (5–10)	6 (5–9)	7 (5–16)	6 (4–18)

Data are shown as mean ± SD or median (minimum–maximum). Age at death is shown in years, disease duration in months and post-mortem interval in hours. LPC stage is shown numerically; 0, non-αSyn; 1, olfactory-only; 2, amygdala-predominant; 3, brainstem-predominant; 4, limbic; 5, neocortical.

*ALS* amyotrophic lateral sclerosis, *FTLD* frontotemporal lobar degeneration, *LB* Lewy bodies, *LPC* Lewy pathology consensus criteria, *MS* multiple sclerosis, *MSA-c* multiple system atrophy cerebellar variant, *MSA-p* multiple system atrophy predominant parkinsonism.

### Morphological appearance of α-synucleinopathy in the retina and optic nerve

**Fig. 1:**
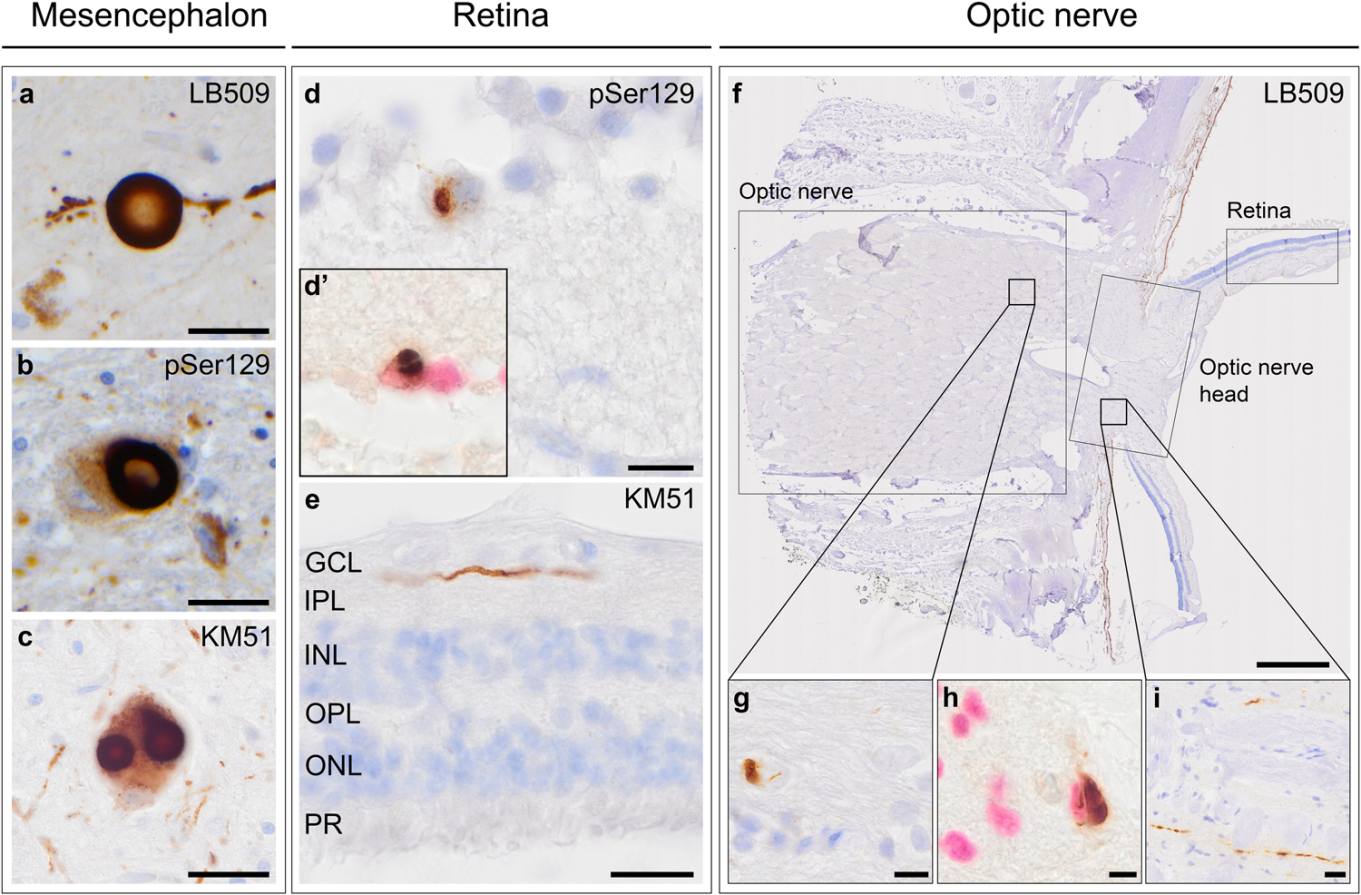
Localisation and morphology of αSyn pathology in the retina and optic nerve. Mesencephalon of a Parkinson’s disease case was used as a positive control, here showing immunostaining with **a** LB509, **b** pSer129 and **c** KM51. **d** pSer129 showed a neuronal staining in a ganglion cell located in the GCL of the retina of a Parkinson’s disease dementia case. **d’** αSyn cytoplasmic inclusion (brown) in a ganglion cell was also observed with a nuclear staining for ganglion cells using NeuN (Liquid Permanent Red). **e** Lewy-like neurites were observed in the IPL of a Parkinson’s disease dementia case. **f**–**h** The optic nerve showed αSyn cytoplasmic inclusions in multiple system atrophy (#54). **h** αSyn cytoplasmic inclusions were observed in oligodendrocytes by co-labelling with anti-SOX-10, which labels nuclei of oligodendrocytes (Liquid Permanent Red). **i** Multiple system atrophy cases additionally showed Lewy-like neurites in the optic nerve head (#54). Immunostaining is shown with DAB (brown), and nuclei are counterstained with haematoxylin (blue). In (**d’**) and (**h**), nuclei are immunolabelled and stained with Liquid Permanent Red. Scale bars: **a**–**c**, **e**, **i** = 20 µm, **d**, **g** = 10 µm, **f** = 800 µm, **h** = 5 µm. GCL ganglion cell layer, IPL inner plexiform layer, INL inner nuclear layer, OPL outer plexiform layer, ONL outer nuclear layer, PR photoreceptors.

Mesencephalon tissue of a Parkinson’s disease case was immunostained with LB509 (Fig. 1a), pSer129 (Fig. 1b) and KM51 (Fig. 1c) as a positive control. In three α-synucleinopathy cases (#35, #36, #42), immunostaining with pSer129 and LB509 showed a sporadic cytoplasmic inclusion in cells within the ganglion cell layer (Fig. 1d), which was also visualised using anti-NeuN (Fig. 1d’). Lewy-like neurites were seen in the retinal nerve fibre layer and inner plexiform layer of the retina (Fig. 1e) as assessed with LB509, pSer129 and KM51. No visual differences were observed in the appearance of Lewy-like neurites using these three antibodies. Immunohistochemistry with all three antibodies also revealed pathological structures in the optic nerve (Fig. 1f). Here, αSyn pathology was mainly observed as cellular inclusions, specifically in cases with multiple system atrophy resembling GCIs (Fig. 1g), which was also shown by double-labelling with a marker for oligodendrocytes using anti-SOX10 (Fig. 1h). Optic nerve tissue showed αSyn pathology resembling GCIs in all six multiple system atrophy cases, as opposed to other cases with optic nerve tissue (*n* = 22). Moreover, although all multiple system atrophy cases had additional Lewy body brain pathology, only two of them (#54, #55) also showed neurites in the optic nerve head at the level of the lamina cribrosa (Fig. 1f, i). In contrast, none showed pathology resembling Lewy neurites in the retina.

### Specific occurrence of αSyn pathology in the retina and optic nerve in synucleinopathies

**Fig. 2:**
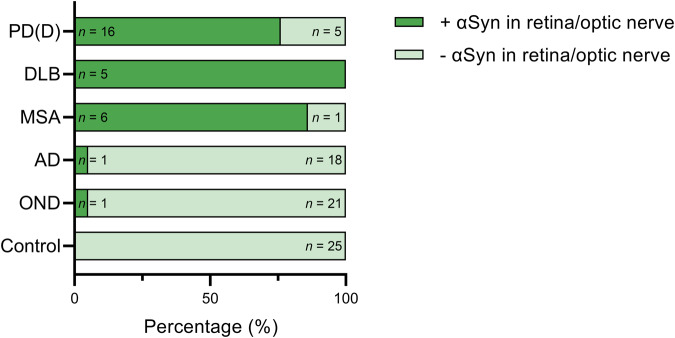
Presence of retinal/optic nerve αSyn pathology in different clinicopathological subgroups. Shown is the percentage of cases with αSyn pathology (dark green indicates presence, light green indicates absence) in the retina/optic nerve per subgroup. The highest percentage of cases with αSyn pathology in the retina/optic nerve is seen in dementia with Lewy bodies (DLB) (100%), followed by multiple system atrophy (MSA) (86%) and Parkinson’s disease (dementia) (PD(D)) (76%). Additionally, one Alzheimer’s disease (AD) and one FTLD-tau (OND) case, both with substantial αSyn pathology in the brain, showed αSyn pathology in the retina/optic nerve. Of the Parkinson’s disease (dementia) cases, 18% had no αSyn pathology in the retina or optic nerve. One multiple system atrophy case had no optic nerve tissue available (#58). This case showed no αSyn pathology in the retina, similar to the other multiple system atrophy cases. PD(D) Parkinson’s disease (dementia), DLB dementia with Lewy bodies, MSA multiple system atrophy, AD Alzheimer’s disease, OND other neurodegenerative diseases, *n* number.

αSyn pathology was assessed in the retina and optic nerve in different clinicopathological groups (Fig. 2). For this analysis, αSyn pathology in the retina and optic nerve was combined, resulting in a total score to gain an inclusive interpretation of the pathologic manifestation per case (Supplementary Table 2). αSyn pathology in the retina/optic nerve was primarily seen in cases with a primary α-synucleinopathy (Fig. 2). The highest ratio was seen for dementia with Lewy bodies (5/5), followed by multiple system atrophy (6/7) and Parkinson’s disease (dementia) (16/21). Also, one Alzheimer’s disease (1/19; #65) and one frontotemporal lobar degeneration-tau case (1/22; #83) showed αSyn pathology in the form of Lewy neurites in the retina/optic nerve, both of which had substantial αSyn co-pathology in the brain (Braak LB stage IV and III, respectively). None of the non-neurological controls (0/25) showed αSyn pathology in the retina/optic nerve. Although a large proportion of Parkinson’s disease (dementia) cases showed corresponding αSyn pathology in the retina/optic nerve, four out of 11 Parkinson’s disease cases (#27, #28, #31, #45) and one out of 10 Parkinson’s disease dementia cases (#29) did not. On a group level, a significant association was found between αSyn pathology in the retina/optic nerve and primary α-synucleinopathies (i.e. Lewy body disease and multiple system atrophy), where the highest odds ratio was found for multiple system atrophy (*P* = 0.001; Table 2). Calculating the accuracy of αSyn pathology in the retina/optic nerve for identifying the presence of a primary α-synucleinopathy yielded a sensitivity of 82% and specificity of 97%, with a positive predictive value of 93% and a negative predictive value of 91%.

**Table 2:** Univariable logistic regression analyses predicting the likelihood of αSyn pathology in the retina/optic nerve.

Statistical analysis was performed using univariable binary logistic regression. The Forest plot graphs the odds ratio with the 95% confidence intervals of the different variables. The axis scale is logarithmic with log-spaced ticks.

*LB* Lewy body.

^1^Other neurodegenerative diseases group is used as reference.

^2^Braak LB 0-III is used as reference.

**Fig. 3:**
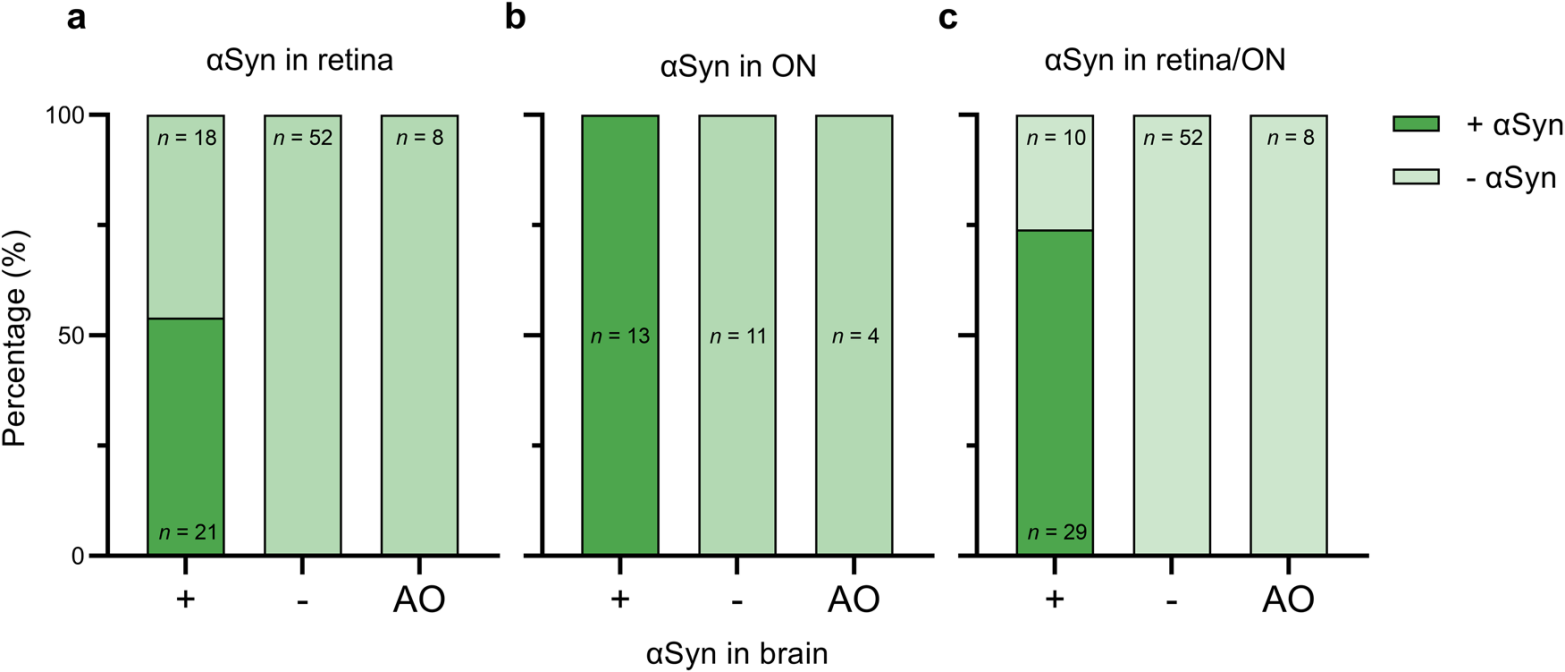
Presence of αSyn pathology in the retina and optic nerve in cases with αSyn pathology in the brain, cases without αSyn pathology in the brain and amygdala-only αSyn pathology. Shown is the percentage of cases with αSyn pathology (dark green indicates presence, light green indicates absence) in **a** retina, **b** optic nerve and **c** retina/optic nerve combined in cases with (+), without (−) and amygdala-only (AO) αSyn pathology in the brain. **a** 54% of the cases with αSyn pathology in the brain (+) showed αSyn pathology in the retina versus 0% of the cases without αSyn pathology in the brain (*−*) or with amygdala-only (AO) αSyn pathology. **b** Of cases with optic nerve tissue available (*n* = 28), all clinicopathological α-synucleinopathies (+; *n* = 13) showed αSyn pathology in the optic nerve as opposed to none of the other cases (*n* = 15). **c** Of the cases with αSyn in the brain ranging from brainstem-predominant to neocortical, 74% showed αSyn pathology in the retina/optic nerve. All cases without αSyn in the brain (−; *n* = 52) showed the absence of αSyn pathology in the retina/optic nerve, as did the cases with amygdala-only αSyn pathology (AO; *n* = 8). ON optic nerve, AO amygdala-only, *n* number.

Further exploring the relation between αSyn pathology in the retina and optic nerve versus the presence of αSyn pathology in the brain, the optic nerve was found to be more accurately associated with the presence of αSyn in the brain than the retina (negative predictive value of 100% for the optic nerve versus 74% for the retina), as is shown in Fig. 3. As depicted here, 54% of the cases with αSyn pathology in the brain showed αSyn pathology in the retina (Fig. 3a) versus 100% in the optic nerve (Fig. 3b). When dichotomous scores of the retina and optic nerve were combined, 74% of the cases with αSyn pathology in the brain also showed αSyn pathology in the retina/optic nerve (Fig. 3c). None of the amygdala-only cases showed αSyn pathology in the retina or optic nerve. In addition, none of the cases without αSyn in the brain (*n* = 52) showed αSyn pathology in the retina or optic nerve, indicating a high specificity.

### αSyn pathology in the retina/optic nerve correlates with αSyn pathology in the brain

**Fig. 4:**
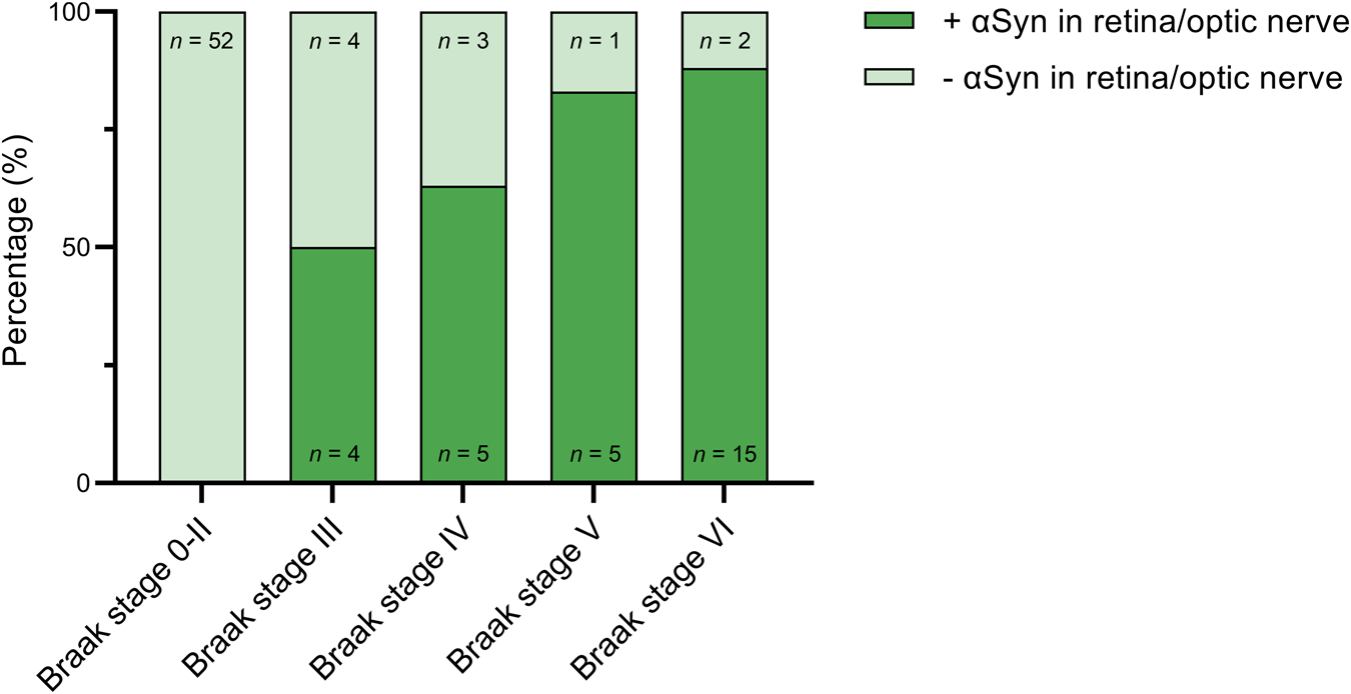
Relation between αSyn pathology in the retina/optic nerve and Braak LB stage. Shown is the percentage of cases with αSyn pathology (dark green indicates presence, light green indicates absence) in the retina/optic nerve in different Braak stages. αSyn pathology in the retina/optic nerve was not observed in cases with lower Braak stages ranging from 0 to II. With increasing Braak stage, αSyn pathology in the retina/optic nerve gradually increased and was most frequently seen in cases with Braak stage V/VI.

**Fig. 5:**
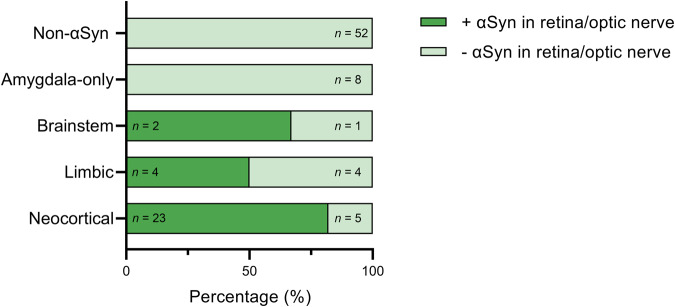
Relation between αSyn pathology in the retina/optic nerve and the LPC staging system. Shown is the percentage of cases with αSyn pathology (dark green indicates presence, light green indicates absence) in the retina/optic nerve in different stages of α-synucleinopathy according to the LPC staging system. αSyn pathology in the retina/optic nerve was absent in cases without αSyn in the brain (non-αSyn) and with amygdala-only αSyn presence. Cases that did have αSyn pathology in the retina/optic nerve had an LPC stage of brainstem-predominant (67%), limbic (50%) or neocortical (82%). LPC Lewy pathology consensus criteria.

Whether there was a significant association between αSyn pathology in the retina/optic nerve and the spread of αSyn pathology in the brain was analysed using the Braak (Fig. 4) and LPC staging systems (Fig. 5). Logistic regression analysis indicated that the likelihood of finding αSyn pathology in the retina/optic nerve increased with Braak stage with the highest odds ratio for stage VI (*P* < 0.001) (Table 2). Notably, eight cases had amygdala-only αSyn presence and could not be classified within the Braak staging system. The occurrence of αSyn pathology within the different stages of LPC is shown in Fig. 5, illustrating the absence of αSyn pathology in the retina/optic nerve in cases without αSyn presence in the brain and in cases with amygdala-only αSyn presence. Because no data was available on cases with olfactory-only αSyn presence, and none of the amygdala-only cases showed αSyn pathology in the retina or optic nerve, logistic regression analysis could not assess the association with the LPC staging system.

In addition, the relation between clinical variables and the presence of αSyn pathology in the retina/optic nerve was studied (Table 2). Corrected for age at death, sex and αSyn in the brain, visual hallucinations were significantly related to αSyn pathology in the retina/optic nerve (*P* = 0.02), whereas dementia and disease duration were not.

## Discussion

Developing retinal biomarkers for various neurodegenerative diseases requires understanding of the retina and optic nerve pathology and the connection with neuropathology in the brain. In this post-mortem study, the prevalence of αSyn pathology in the retina and optic nerve was assessed in a well-characterised cohort with different neurodegenerative diseases and non-neurological controls. αSyn pathology was mainly observed in the retina and optic nerve of primary α-synucleinopathies as opposed to other neurodegenerative disorders and controls without αSyn in the brain. There is a high specificity (97%) and sensitivity (82%) for the presence of αSyn in the retina and optic nerve in relation to brain αSyn pathology as part of a primary α-synucleinopathy. Results from this study suggest that αSyn pathology in the retina and optic nerve is associated with the distribution of αSyn pathology according to the Braak LB stage and thus mirrors the pathological presence of αSyn in the brain.

The observation of αSyn pathology in the retina and optic nerve tissue of cases with α-synucleinopathies is in concordance with previous studies^7–9^. In the present study, αSyn pathology was observed as Lewy neurites in the retina and optic nerve and as GCIs in the optic nerve. Sporadically and only in primary synucleinopathy cases, cytoplasmic inclusions were observed in the ganglion cell layer, presumably in ganglion cells. These structures resemble what was earlier described as Lewy body-like inclusions^9^. However, retinal cytoplasmic inclusions observed in the present study did not show similarity with the classic halo-morphology seen in the brain, nor were they similar in size. The absence of classic Lewy bodies in this study suggests that the retina differs from the brain in response mechanisms to αSyn-associated pathology and may hold essential qualities such as a highly metabolic environment aimed at preventing the formation of protein aggregation until the stage of classic inclusion bodies^23^.

Although previous studies^7–10^^,21,24^, reported on the presence and characterisation of αSyn pathology in the retina in α-synucleinopathies, αSyn pathology in multiple system atrophy had not yet been described. Multiple system atrophy cases showed αSyn pathology in the form of cytoplasmic inclusion bodies resembling GCIs in the optic nerve, which were not observed in other cases with optic nerve tissue available. In-vivo optical coherence tomography data support optic nerve pathology in multiple system atrophy. In their review, Mendoza-Santiesteban and colleagues^25^ outlined data on patients with multiple system atrophy that show a specific pattern of damage of the post-laminar myelinated optic nerve axons. They describe damaging of the M-cell axons arising from the peripheral retina and the sparing of the P-cell axons originating from the macular area. This leads to retinal nerve fibre layer thinning around the optic nerve head in the superior, nasal and inferior quadrant, but not in the temporal quadrant, as opposed to the more general retinal nerve fibre layer thinning seen in Parkinson’s disease cases. The high specificity and sensitivity found in the present study for GCI pathology in the optic nerve with relation to the presence of multiple system atrophy-associated brain pathology, and the absence of Lewy pathology in the retinas of these cases, makes the eye an exciting candidate in the search for biomarkers that can discriminate between α-synucleinopathies.

We found a clear relation between Lewy pathology in the retina/optic nerve and αSyn pathology in the brain. Nonetheless, in 18% of the primary α-synucleinopathy cases, no αSyn pathology in the retina or optic nerve was observed. One explanation for the absence of αSyn pathology could be that pathology was missed due to a Type II error in assessing cross-sections. On the other hand, the lack of αSyn pathology may suggest that the neuronal tissue of the eye is differently involved within the hierarchical spreading of αSyn pathology in the brain. In a recent review by Jellinger^26^, evidence was outlined for multiple routes of disease progression that are different from Braak’s proposed caudorostral pathway^27^, and that pathological progression may vary between subtypes. The pathological variability of ophthalmologic αSyn seen in this study could be explained by this heterogeneity among α-synucleinopathies. The significant association with the Braak LB staging system suggests that αSyn pathology in the retina/optic nerve does depend on localisation and/or load of αSyn pathology in the brain. In particular, the strong association with stage VI and the high proportion of cases with αSyn pathology in the retina/optic nerve within the neocortical LPC stage suggests that αSyn pathology in the retina/optic nerve may predict neocortical αSyn involvement.

Ophthalmologic αSyn was also present in one Alzheimer’s disease and one frontotemporal lobar degeneration-tau case, showing substantial αSyn pathology in the brain. More interestingly, all Alzheimer’s disease cases with amygdala-predominant αSyn presence did not show αSyn pathology in the retina or optic nerve. A recent post-mortem study demonstrates distinct distribution patterns of Lewy body pathology in the presence of Alzheimer’s disease^28^. The authors state that amygdala-predominant Lewy bodies are Alzheimer’s disease interacting pathologies as opposed to brainstem and limbic Lewy bodies. This would explain the absence of αSyn pathology in the retina and optic nerve of cases with amygdala-only αSyn pathology and suggests that the eye has a disease-specific involvement in the spread of αSyn pathology of the brain.

Interestingly, a significant association was observed between αSyn pathology in the retina/optic nerve and visual hallucinations. Given the phenomenologically complex nature of the hallucinations of subjects in this cohort, it is likely that the underlying cause lies in the presence of cortico-cerebral αSyn pathology rather than ophthalmologic αSyn pathology^29^. To understand the association between complex visual hallucinations as seen in Lewy body disease and αSyn pathology in the retina and optic nerve, future studies should focus on the possibility of hierarchical spreading of αSyn pathology along the retino-geniculo-cortical pathway^30,31^.

A strength of this study is that multiple antibodies detecting different epitopes were used to asses ophthalmologic αSyn pathology. Additionally, besides the retina, also optic nerve tissue was assessed. Data from this study indicate that αSyn pathology is more prominent in the optic nerve and optic nerve head than in the retina. Therefore, future studies should focus on exploring the optic nerve head, including the lamina cribrosa in addition to the retina, to develop methods for detecting αSyn pathology using in-vivo scanning modalities. A limitation of this study is that only cross-sections and not whole-mount retinas were assessed, and pathology could have been missed, potentially causing a Type II error.

In conclusion, this study on a mixed cohort of neurodegenerative diseases and non-neurological controls demonstrates that the detection of αSyn pathology in the retina and optic nerve shows a high specificity (97%) and sensitivity (82%) regarding the presence of primary α-synucleinopathy of the brain. αSyn pathology in the retina and optic nerve correlates with Lewy body disease and multiple system atrophy as opposed to Alzheimer’s disease and other neurodegenerative diseases and is associated with higher Braak LB stages. Here, we report that the hallmark GCI pathology observed in the brain in cases of multiple system atrophy is also detected in the optic nerve. The absence of Lewy pathology in the retina in multiple system atrophy could contribute to developing an in-vivo biomarker discriminating between multiple system atrophy and other α-synucleinopathies. The results of this study support the development of retinal biomarkers for neurodegenerative diseases such as α-synucleinopathies.

## Methods

### Post-mortem tissue

Post-mortem eyes and brain tissue of 99 donors were collected from 2009 until 2022 by the Netherlands Brain Bank^32^ (Amsterdam, The Netherlands, https://www.brainbank.nl). The VUmc medical ethics committee approved the Netherlands Brain Bank donor programme (reference# 2009/148). All donors consented in writing to use their tissue and clinical records for research purposes in compliance with ethical standards. Brain autopsies were performed according to the Code of Conduct of Brain Net Europe^33^.

Brain autopsy was performed within 12 h post-mortem, after which brain tissue was formalin-fixed (10%; 4 weeks) and embedded in paraffin. Neuropathological diagnosis was performed (AR) according to the guidelines of the BrainNet Europe Consortium^34^, including assessment of Braak-stage for Lewy bodies (Braak LB stage)^27^ and Alzheimer’s disease neuropathological changes^35^, including Thal-phase for Aβ^36,37^, Braak-stage for NFTs^38^ and CERAD score for neuritic plaque pathology^39^. Cases were grouped according to clinicopathological diagnosis in Parkinson’s disease (and dementia)^40,41^ (*n* = 21), dementia with Lewy bodies (*n* = 5)^42^, Multiple system atrophy (*n* = 7), Alzheimer’s disease^37^ (*n* = 19), other neurodegenerative diseases^43–45^ (*n* = 22) and non-neurological controls (*n* = 25). During screening, 12 cases were found to have a medical history of ophthalmologic disease, including glaucoma (*n* = 5), age-related macular degeneration (*n* = 3), optic neuritis (*n* = 1), retinal detachment (*n* = 2) and macular pucker (*n* = 1). This was, however, not an exclusion criterium for this study. Cohort characteristics are shown in Supplementary Table 1.

### Post-mortem retinal tissue preparation

Post-mortem enucleated eyes were directly collected in 4% paraformaldehyde (PFA) and stored for 48 h before further processing (Supplemental Table 1). Some eyes were initially cryopreserved and stored for later use. In short, these eyes were frozen using isopentane at −90 °C and stored at −80 °C. To prevent damage to the retina caused by the pressure of frozen tissue, the cornea and lens were removed before freezing, which allowed for the expansion of vitreous humour. Consequently, tissue-tek O.C.T. compound (Sakura, Tokyo, Japan) was used to fill the eyeball to compensate for vitreous humour loss, to avoid folding of the eyeball during the freezing process, and to prevent direct contact between the retinal tissue and isopentane. Isopentane, an effective cryoprotectant, prevented cryo-artefacts from ice crystal formation during freezing. Frozen eyes were defrosted at room temperature (RT) in PFA for 48 h before dissection. PFA fixed eyes were dissected through the horizontal and vertical axis, resulting in temporal–superior, temporal–inferior, nasal–superior and nasal–inferior quadrants as previously described^4,5^.

### Immunohistochemistry

Tissue sections (10 μm thickness) from formalin-fixed, paraffin-embedded retina of the superior and inferior axis were sequentially mounted on TOMO glass slides (Matsunami, Osaka, Japan). Brain tissue sections (7 μm thickness) were mounted on Superfrost plus glass slides (VWR, Pennsylvania, USA). In all cases, according to the protocol of the BrainNet Europe Consortium^34^, the cortico-cerebral presence of αSyn pathology immunostained with KM51 against full-length αSyn (Novocastra/NCL-ASYN, clone KM51, RRID: AB_442103) was assessed in the brainstem (mesencephalon, medulla oblongata, pontine tegmentum, substantia nigra) and limbic system (amygdala, middle hippocampus at the geniculate body). Clinicopathological suspected α-synucleinopathies were additionally assessed in the neocortex (temporal pole, medial frontal gyrus, inferior parietal lobe, occipital pole V1/V2). Sections were air-dried overnight at 37 °C before staining. Sections were deparaffinised and rehydrated using sequential incubations in xylene, alcohol and water. Endogenous peroxidase activity was suppressed by incubating the sections with 0.3% H_2_O_2_ in phosphate buffer saline (PBS; pH 7.4) for 30 min. Antigen retrieval was performed in 10 mM/L citrate buffer (pH 6.0) and heated using an autoclave (20 min at 121 °C). Retina sections immunostained with KM51 (dilution 1:100) and LB509 (epitope from Lewy bodies purified from DLB patients, Invitrogen, 1:500, RRID: AB_2920932) were additionally pre-treated with 100% formic acid (10 min at RT) and washed with PBS. Sections were incubated overnight at RT with primary antibodies LB509, KM51 and anti-αSyn pSer129^46^ (ABCAM, Cambridge, UK, 1:100, RRID: AB_869973) diluted in antibody diluent (Sigma-Aldrich, Saint Louis, USA). Omission of the primary antibody was used as a negative control, and mesencephalon tissue from a Parkinson’s disease case was used as a positive control. After primary antibody incubation, the sections were washed with PBS, incubated with anti-mouse/rabbit HRP Envision (DAKO, Glostrup, Denmark) (30 min) and subsequently washed using PBS. 3,3’-Diaminobenzine (DAB; DAKO) was used as a chromogen for colour development (5 min at RT). Sections were counterstained with haematoxylin, dehydrated using alcohol and xylene and mounted using Quick-D (Klinipath; Duiven, The Netherlands).

### Immunohistochemical double labelling

Tissue sections were stained for the αSyn markers (LB509, KM51 and pSer129) as described above. After DAB treatment, antibody complexes were removed by heating the sections in citrate buffer using a microwave (10 min, close to boiling point). The tissue sections were incubated with either anti-SOX10 (Cell Marque 1:100, clone EP268, RRID: 2941085) or anti-NeuN (Abcam, 1:500, RRID: AB_2532109) for 1 h at room temperature. An alkaline phosphatase (AP)-conjugated goat anti-rabbit Ig (SouthernBiotech 1:250, RRID: AB_2722612) was incubated for 1 h, and colour development was performed using Liquid Permanent Red (DAKO). Tissue sections were mounted using Aquatex aqueous mounting medium (Merck, Darmstadt, Germany).

### Assessment and quantification of immunoreactivity

The presence and localisation of αSyn pathology were assessed in 99 donors. Per case, a total of four sections were immunostained: three sections of the superior retina immunostained with LB509, pSer129 and KM51, and one section of the inferior retina using LB509 (Supplementary Fig. 1). For 28 donors, optic nerve tissue was available, which was separately assessed for αSyn pathology. Immunostained cross-sections were imaged using an Olympus VS200 slide scanner. With the assessment of LB509, pSer129 and KM51 in the superior retina, LB509 appeared most sensitive in detecting αSyn pathological structures (Supplementary Fig. 1a). For this reason and to reduce the chance of sampling bias, the inferior retina was stained with LB509 (Supplementary Fig. 1d). Overall, minor variability in the detection of pathological structures in the retina and optic nerve was observed between the different markers. Retinal and optic nerve tissue was assessed based on dichotomised scoring; if Lewy bodies, Lewy neurites or GCIs were observed in at least one of four cross-sections within a case, it was scored as positive (+). When absent in all cross-sections, the case was scored as negative (−) (Supplementary Table 2). In brain tissue, αSyn was assessed using KM51 and immunoreactivity was scored with an Olympus BX41 microscope in different brain regions with total surface areas ranging from 2 to 4 cm^2^ per section. Staging according to the new proposed Lewy pathology consensus criteria (LPC)^47^ was performed by scoring the brain tissue in a dichotomous manner, resulting in “amygdala-predominant” also encompassing cases with “amygdala-only” presence of αSyn^48,49^, “brainstem-predominant”, “limbic” and “neocortical” Lewy pathology. Assessment of αSyn pathology in the brain and the retina/optic nerve was performed separately and masked for diagnosis. All figures were composed with GraphPad Prism (RRID: SCR_002798, version 9.3.1) and Adobe Photoshop (Adobe Systems Incorporated, RRID: SCR_014199, version 23.5).

### Statistical analysis

Demographical differences between diagnostic groups were studied using a one-way ANOVA for continuous data and Chi-square tests for dichotomous data. Univariable binary logistic regression was performed to examine the effect of independent variables on the presence of αSyn pathology in the retina and optic nerve. All analyses were corrected for age at death and sex and were performed using SPSS Statistics (IBM SPSS Statistics, RRID: SCR_016479) version 28. A *P* value of <0.05 was considered significant.

### Supplementary information

Supplementary material

## Data Availability

All data generated or analysed during this study are included (see also Supplementary Information files).
