# Supplementary material for: α-Synuclein pathology in post-mortem retina and optic nerve is specific for α-synucleinopathies
Source: NPJ Parkinsons Dis. 2023 Aug 28;9:124. doi: 10.1038/s41531-023-00570-5 (PMC10462645; doi:10.1038/s41531-023-00570-5)
Supplement: Supplementary file 1 — Supplementary material [file 41531_2023_570_MOESM1_ESM.pdf]

Supplementary Figure 1 Detection of αSyn pathology in the retina/optic nerve by individual antibodies and regions.

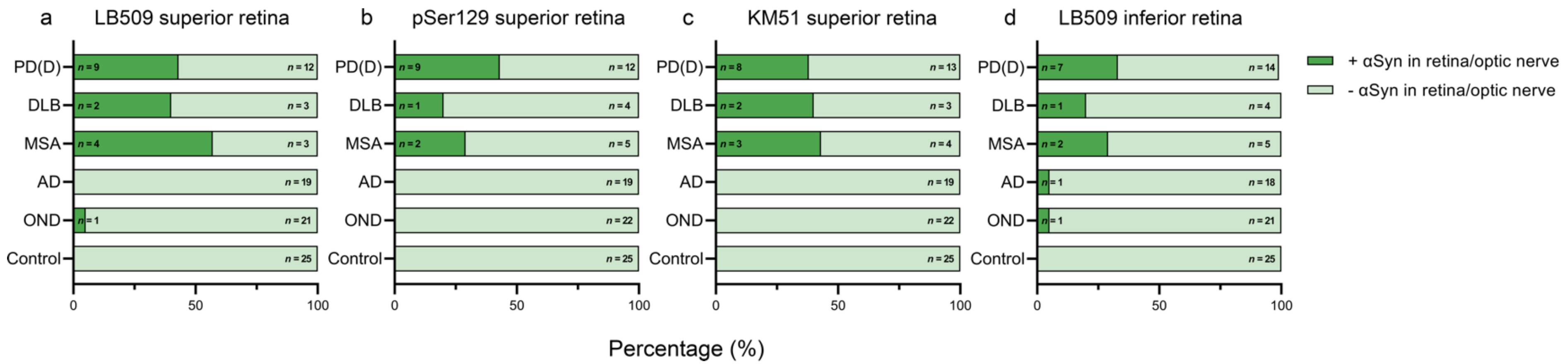

Supplementary Figure 1 Detection of αSyn pathology in the retina/optic nerve by individual antibodies and regions. Immunohistochemistry was performed with different antibodies (LB509, pSer129 and KM51) on different cross-sections of the **(a-c)** superior and **(d)** inferior part of the retina. Shown is the percentage of αSyn pathology (dark green indicates presence, light green indicates absence) in the retina/optic nerve. Because LB509 seemed the most sensitive marker in detecting αSyn pathological structures in the superior retina, the inferior retina was also stained with this marker. Overall, minor variability in the detection of pathological structures in the retina and optic nerve was observed between the different markers and between the superior and inferior retina.

Supplementary Table 1 Cohort characteristics of this study

Case number	Sex	Age at death	Cause of death	PMI	Fixation	Ophthalmologic history	Visual hallucinations	Dementia	Disease duration	Braak NFT stage	Thal Aβ phase	CERAD score	CAA type/stage
1	F	33	Euthanasia	07:30	PFA	none	-	-	n/a	0	0	0	none
2	F	47	Euthanasia	06:05	Frozen / PFA	none	-	-	n/a	0	0	0	none
3	M	49	Euthanasia	06:15	Frozen / PFA	none	-	-	n/a	0	0	0	none
4	F	55	Suicide by medication	09:50	Frozen / PFA	none	-	-	n/a	0	0	0	none
5	F	63	Metastatic breast cancer	05:00	Frozen / PFA	none	-	-	n/a	0	0	0	none
6	M	68	Sudden death	08:55	Frozen / PFA	none	-	-	n/a	0	0	0	none
7	F	57	Euthanasia with metastatic urothelial cancer	07:40	Frozen / PFA	none	-	-	n/a	0	0	0	1/1
8	F	60	Euthanasia	05:30	PFA	none	-	-	n/a	0	0	0	2/1
9	F	62	Suicide by strangulation	11:40	Frozen / PFA	Retinal detachement (2007, 2009)	-	-	n/a	I	0	0	none
10	F	63	Euthanasia	07:30	PFA	Acute glaucoma (1990/2015)	-	-	n/a	II	1	0	2/1
11	F	72	Euthanasia	08:45	PFA	none	-	-	n/a	III	1	0	2/1
12	F	76	Hepatic failure and colon carcinoma	07:15	Frozen / PFA	none	-	-	n/a	II	0	0	none
13	F	79	Pneumonia	06:00	PFA	none	-	-	n/a	III	3	2	2/1
14	F	79	Cardiac insufficiency	10:30	Frozen / PFA	none	-	-	n/a	I	0	0	none
15	M	80	Euthanasia	05:10	Frozen / PFA	none	-	-	n/a	II	0	0	none

16	F	80	Severe chronic diarrhea	08:45	PFA	none	-	-	n/a	III	0	0	none
17	F	81	Lymphoplasmatic lymphoma (Kahler)	07:40	PFA	Macular pucker	-	-	n/a	I	2	0	none
18	F	83	Euthanasia	06:50	PFA	none	-	-	n/a	I	3	0	1/3
19	M	87	Pneumonia	03:15	Frozen / PFA	none	-	-	n/a	II	0	0	none
20	M	89	Euthanasia	03:05	PFA	none	-	-	n/a	I	1	0	2/2
21	F	89	Pneumonia and cardiac decompensation	05:40	Frozen / PFA	none	-	-	n/a	II	2	0	2/1
22	M	90	Pneumonia and heart failure	03:55	Frozen / PFA	none	-	-	n/a	II	I	I	none
23	F	98	Heart failure	08:45	PFA	Dry macular degeneration (2010)	-	-	n/a	II	0	0	none
24	M	80	Euthanasia with cholangio carcinoma	04:25	Frozen / PFA	Macular degeneration	-	-	n/a	II	4	0	none
25	F	96	Ceased oral intake	07:30	PFA	none	-	-	n/a	II	1	0	2/1
26	M	65	Intestinal obstruction, ulcerative colitis and severe anemia	05:00	Frozen / PFA	none	-	-	37	I	0	0	none
27	M	80	End stage prostate carcinoma	06:45	PFA	n.a.	n.a.	n.a.	n.a.	II	1	0	2/1
28	F	62	Euthanasia	08:00	Frozen / PFA	none	+	-	300	I	0	0	none
29	M	78	Euthanasia	07:15	PFA	Glaucoma	+	-	204	II	0	0	none
30	F	66	Euthanasia	05:00	Frozen / PFA	Glaucoma (1996), recurrent eye inflammation (syndrome of Cogan), recurrent scleritis (2017), recurrent optic nerve	-	-	192	I	0	0	none

						inflammation (2018)							
31	M	72	Atrial fibrillation	08:45	Frozen / PFA	none	-	-	144	II	1	0	none
32	M	72	Euthanasia		PFA	none	+	-	n.a.	I	1	0	none
33	M	75	Euthanasia	05:50	Frozen / PFA	none	-	-	300	I	2	0	1/1
34	M	77	Euthanasia	04:48	Frozen / PFA	none	-	-	192	I	1	0	none
35	F	79	Euthanasia	03:45	PFA	Glaucoma	+	n.a.	48	II	1	0	none
36	M	75	End stage Parkinson's disease	05:40	PFA	none	+	-	240	II	3	0	1/1
37	M	76	Pneumonia	05:35	Frozen / PFA	none	+	+	60	III	2	1	none
38	M	74	Aspiration pneumonia	08:50	PFA	none	+	+	120	III	2	0	2/1
39	M	81	Euthanasia	08:21	Frozen / PFA	none	+	+	48	III	1	0	1/1
40	F	88	Cardiac cause and viral infection	07:30	PFA	none	+	+	324	III	1	0	none
41	M	67	Dehydration	07:30	Frozen / PFA	none	+	+	180	III	0	0	none
42	M	78	End stage Parkinson's disease	03:30	PFA	none	+	+	240	II	1	0	none
43	F	93	Parkinson's disease and old age	05:40	PFA	none	+	+	300	III	3	1	1/1
44	F	91	Palliative sedation	04:10	Frozen / PFA	none	-	+	144	I	1	0	2/1
45	M	92	Myocardial infarction	10:10	PFA	Dry macular degeneration (2017), vitrectomie OD (2012)	-	-	204	n.a. due to extensive tauopathy	3	1	2/1

46	F	86	Cachexia and dehydration	04:08	Frozen / PFA	none	+	+	168	I	0	0	none
47	M	72	Euthanasia	04:15	Frozen / PFA	none	+	-	48	III	4	2	1/3
48	M	75	Cachexia and dehydration	06:50	PFA	none	+	+	84	I	3	0	2/1
49	F	81	Cachexia	06:30	Frozen / PFA	none	+	-	84	I	1	0	none
50	F	91	Pneumonia by advanced Lewy Body dementia	04:40	Frozen / PFA	none	+	+	72	III	3	1	2/1
51	F	86	Ceased oral intake	10:20	Frozen / PFA	none	+	+	48	IV	5	2	1/2
52	F	72	Unknown cause	08:25	PFA	Glaucoma	-	-	60	II	2	0	none
53	F	57	Euthanasia	05:30	Frozen / PFA	none	-	-	48	I	0	0	none
54	F	59	Pulmonary embolism, pneumonia and myocardial infarction	05:55	Frozen / PFA	none	-	-	36	II	1	0	none
55	M	72	Euthanasia	08:00	PFA	none	-	-	120	I	1	0	1/1
56	F	67	End stage MSA and dehydration	04:00	PFA	none	-	-	60	II	0	0	none
57	M	84	Euthanasia	05:40	PFA	none	-	-	60	IV	?	1	1/2
58	F	76	Euthanasia	04:50	Frozen / PFA	none	-	+	24	IV	3	0	none
59	M	65	Cardiac arrest	07:50	PFA	none	-	+	48	VI	4	3	1/2
60	F	72	End stage Alzheimer's disease	05:05	PFA	none	-	+	120	VI	5	3	2/1
61	M	73	Palliative sedation	07:00	PFA	none	+	+	120	VI	5	3	1/1
62	F	82	Palliative sedation	05:35	Frozen / PFA	none	-	+	60	VI	5	3	1/1
63	M	82	Dysphagia	07:30	PFA	none	-	+	180	VI	5	3	2/1

64	M	84	Euthanasia	05:53	PFA	none	n.a.	n.a.	156	IV	5	2	none
65	M	80	Lobar bleeding	10:50	PFA	none	-	+	120	VI	5	3	1/2
66	F	91	Cerebral vascular incident	06:30	Frozen / PFA	none	-	+	72	V	4	3	1/2
67	F	95	End stage Alzheimer's disease and dehydration	05:30	Frozen / PFA	none	-	+	12	IV	3	2	2/1
68	F	61	Cachexie and dehydration	06:50	PFA	none	-	+	96	VI	5	3	1/1
69	F	66	Sedatives and probably pneumonia	08:00	PFA	none	-	+	120	VI	5	3	2/1
70	F	68	Euthanasia with dementia		PFA	none	-	+	60	V	4	2	2/1
71	M	70	Alzheimer's disease and infection/carcinoma	06:20	Frozen / PFA	none	-	+	120	VI	5	0	none
72	M	71	Multiorgan failure by metastasized pulmonary carcinoma and MS	08:45	Frozen / PFA	none	-	+	312	IV	4	1	none
73	F	73	Euthanasia	05:30	Frozen / PFA	Retinal detachement (year unknown)	-	+	60	VI	5	3	none
74	F	76	Euthanasia	03:45	PFA	none	-	-	144	V	5	3	1/2
75	M	77	Euthanasia	07:00	Frozen / PFA	none	-	+	48	IV	5	2	2/1
76	F	80	Euthanasia	07:05	PFA	none	-	-	84	IV	5	2	2/1
77	F	89	Massive heart attack	05:15	Frozen / PFA	none	-	+	96	IV	3	1	none
78	M	59	Metastatic cancer, unknown primary tumor	06:15	Frozen / PFA	none	-	n.a.	108	I	2	0	2/1
79	F	74	Cachexia	08:10	Frozen / PFA	Posterior vitreous detachment (2007)	-	-	36	I	3	0	none

80	M	65	Infection and dehydration	06:05	Frozen / PFA	none	-	-	156	II	1	0	none
81	F	65	Euthanasia	05:50	Frozen / PFA	none	-	-	84	II	3	1	none
82	F	103	Heart failure	09:50	PFA	none	-	-	n/a	III	3	1	1/1
83	M	71	Sudden death	04:30	PFA	none	-	+	120	II	2	0	none
84	M	65	Pneumonia	05:40	Frozen / PFA	none	-	-	180	n.a. due to extensive tauopathy	1	0	2/1
85	M	80	Respiratory failure due to traumatic cervical paraplegia	09:30	PFA	none	-	-	n/a	II	0	0	none
86	F	95	Ileus caused by tumor	04:21	Frozen / PFA	none	-	-	n/a	III	1	0	2/1
87	M	58	Cardiac arrest	06:00	Frozen / PFA	none	-	+	48	0	0	0	none
88	F	70	Dehydration	05:00	PFA	none	-	+	24	II	1	0	none
89	F	52	Dehydration and cachexia by end stage dementia	08:16	Frozen / PFA	none	-	+	84	I	0	0	none
90	M	75	Pneumonia	17:35	Frozen / PFA	none	-	+	60	II	3	0	none
91	F	74	Aspiration with hypoxemia	05:10	Frozen / PFA	none	-	+	36	II	1	0	none
92	M	65	End stage FTD	05:45	Frozen / PFA	none	-	-	24	II	1	0	1/1
93	F	51	End stage FTD and dyspnea	06:00	PFA	none	+	+	48	0	1	0	none
94	M	50	Unknown cause	06:55	Frozen / PFA	none	-	+	72	0	0	0	none
95	M	71	Euthanasia	06:45	Frozen / PFA	none	-	-	144	I	1	0	none
96	M	54	Euthanasia	06:40	Frozen / PFA	none	-	-	180	I	0	0	none

97	M	84	Unknown cause	09:50	PFA	none	-	-	n/a	II	1	0	1/1
98	M	80	Fever without focus	08:30	PFA	none	+	+	84	II	2	0	none
99	F	70	End stage dementia	07:45	PFA	none	-	+	n/a	II	0	0	none

Abbreviations: CAA, cerebral amyloid angiopathy; FTD, frontotemporal dementia; NFT, neurofibrillary tangles; OD, oculus dexter; PFA, paraformaldehyde; PMI, post-mortem interval; n/a, not applicable; n.a., not available; +, present; -, absent.

Supplementary Table 2 Dichotomized score of αSyn presence in brain, retina and optic nerve tissue

					α-syn in brain			αSyn in retina	αSyn in ON	Retina and ON
**Case number**	**(Clinico)pathological diagnosis**	**Group diagnosis**	**Sex**	**Age at death**	**Binary score**	**Braak LB stage**	**LPC stage**	**Binary score**	**Binary score**	**Total score**
1	CN	CN	F	33	-	0	n/a	-	n.a.	-
2	CN	CN	F	47	-	0	n/a	-	n.a.	-
3	CN	CN	M	49	-	0	n/a	-	-	-
4	CN	CN	F	55	-	0	n/a	-	n.a.	-
5	CN	CN	F	63	-	0	n/a	-	-	-
6	CN	CN	M	68	-	0	n/a	-	n.a.	-
7	CN	CN	F	57	-	0	n/a	-	n.a.	-
8	CN	CN	F	60	-	0	n/a	-	n.a.	-
9	CN	CN	F	62	-	0	n/a	-	-	-
10	CN	CN	F	63	-	0	n/a	-	-	-
11	CN	CN	F	72	-	0	n/a	-	n.a.	-
12	CN	CN	F	76	-	0	n/a	-	n.a.	-
13	CN	CN	F	79	-	0	n/a	-	n.a.	-
14	CN	CN	F	79	-	0	n/a	-	n.a.	-
15	CN	CN	M	80	-	0	n/a	-	-	-
16	CN	CN	F	80	-	0	n/a	-	-	-
17	CN	CN	F	81	-	0	n/a	-	-	-

18	CN	CN	F	83	-	0	n/a	-	n.a.	-
19	CN	CN	M	87	-	0	n/a	-	n.a.	-
20	CN	CN	M	89	-	0	n/a	-	-	-
21	CN	CN	F	89	-	0	n/a	-	n.a.	-
22	CN	CN	M	90	-	0	n/a	-	n.a.	-
23	CN	CN	F	98	-	0	n/a	-	n.a.	-
24	CN	CN	M	80	+	n/a	Amygdala predominant	-	n.a.	-
25	CN	CN	F	96	+	4	Limbic	-	n.a.	-
26	PD	PD(D)	M	65	+	3	Limbic	+	n.a.	+
27	PD	PD(D)	M	80	+	3	Brainstem predominant	-	n.a.	-
28	PD	PD(D)	F	62	+	4	Neocortical (amygdala predominant)	-	n.a.	-
29	PDD	PD(D)	M	78	+	6	Neocortical (amygdala predominant)	-	n.a.	-
30	PD	PD(D)	F	66	+	6	Neocortical	+	n.a.	+
31	PD	PD(D)	M	72	+	6	Neocortical	-	n.a.	-
32	PD	PD(D)	M	72	+	6	Neocortical	+	+	+
33	PD	PD(D)	M	75	+	6	Neocortical	+	n.a.	+
34	PD	PD(D)	M	77	+	6	Neocortical	+	+	+
35	PD	PD(D)	F	79	+	5	Neocortical	-	+	+
36	PD	PD(D)	M	75	+	6	Neocortical	+	n.a.	+
37	PDD	PD(D)	M	76	+	6	Neocortical	+	n.a.	+

38	PDD	PD(D)	M	74	+	6	Neocortical	+	n.a.	+
39	PDD	PD(D)	M	81	+	6	Neocortical	+	n.a.	+
40	PDD	PD(D)	F	88	+	5	Neocortical	+	+	+
41	PDD	PD(D)	M	67	+	6	Neocortical	+	n.a.	+
42	PDD	PD(D)	M	78	+	6	Neocortical	+	n.a.	+
43	PDD	PD(D)	F	93	+	6	Neocortical	+	n.a.	+
44	PDD	PD(D)	F	91	+	4	Neocortical	+	n.a.	+
45	PD + PSP	PD(D)	M	92	+	4	Neocortical	-	n.a.	-
46	PDD + PSP	PD(D)	F	86	+	5	Neocortical	+	+	+
47	DLB	DLB	M	72	+	6	Neocortical	+	n.a.	+
48	DLB	DLB	M	75	+	6	Neocortical	-	+	+
49	DLB	DLB	F	81	+	4	Neocortical	+	n.a.	+
50	DLB	DLB	F	91	+	6	Neocortical	+	n.a.	+
51	DLB(/AD)	DLB	F	86	+	6	Neocortical	+	n.a.	+
52	MSA-p	MSA	F	72	+	3	Brainstem predominant	-	+	+
53	MSA-p	MSA	F	57	+	4	Limbic	-	+	+
54	MSA-p	MSA	F	59	+	3	Limbic	-	+	+
55	MSA-p	MSA	M	72	+	5	Neocortical	-	+	+
56	MSA-c/p	MSA	F	67	+	5	Neocortical	-	+	+
57	MSA-c/p	MSA	M	84	+	4	Neocortical	-	+	+

58	MSA-c/p	MSA	F	76	+	5	Neocortical	-	n.a.	-
59	AD	AD	M	65	+	n/a	Amygdala predominant	-	n.a.	-
60	AD	AD	F	72	+	n/a	Amygdala predominant	-	-	-
61	AD	AD	M	73	+	n/a	Amygdala predominant	-	-	-
62	AD	AD	F	82	+	n/a	Amygdala predominant	-	-	-
63	AD	AD	M	82	+	n/a	Amygdala predominant	-	-	-
64	AD	AD	M	84	+	3	Limbic	-	n.a.	-
65	AD	AD	M	80	+	4	Limbic	+	n.a.	+
66	AD	AD	F	91	+	3	Limbic	-	n.a.	-
67	AD	AD	F	95	+	3	Limbic	-	n.a.	-
68	AD	AD	F	61	-	0	n/a	-	n.a.	-
69	AD	AD	F	66	-	0	n/a	-	n.a.	-
70	AD	AD	F	68	-	0	n/a	-	-	-
71	AD	AD	M	70	-	0	n/a	-	n.a.	-
72	AD	AD	M	71	-	0	n/a	-	-	-
73	AD	AD	F	73	-	0	n/a	-	n.a.	-
74	AD	AD	F	76	-	0	n/a	-	n.a.	-
75	AD	AD	M	77	-	0	n/a	-	n.a.	-
76	AD	AD	F	80	-	0	n/a	-	n.a.	-
77	AD	AD	F	89	-	0	n/a	-	n.a.	-

78	CBD	OND	M	59	-	0	n/a	-	n.a.	-
79	CBD	OND	F	74	-	0	n/a	-	n.a.	-
80	PSP	OND	M	65	-	0	n/a	-	n.a.	-
81	PSP	OND	F	65	+	n/a	Amygdala predominant	-	n.a.	-
82	ARTAG	OND	F	103	+	n/a	Amygdala predominant	-	n.a.	-
83	FTLD-tau	OND	M	71	+	3	Brainstem predominant	+	+	+
84	FTLD-tau	OND	M	65	-	0	n/a	-	n.a.	-
85	ARTAG + PART	OND	M	80	-	0	n/a	-	n.a.	-
86	ARTAG + PART	OND	F	95	-	0	n/a	-	n.a.	-
87	FTLD-TDP type A	OND	M	58	-	0	n/a	-	n.a.	-
88	FTLD-TDP type A	OND	F	70	-	0	n/a	-	n.a.	-
89	FTLD TDP type B	OND	F	52	-	0	n/a	-	n.a.	-
90	FTLD-TDP type B	OND	M	75	-	0	n/a	-	-	-
91	FTLD-TDP type B	OND	F	74	-	0	n/a	-	n.a.	-
92	FTLD-TDP type B / ALS	OND	M	65	-	0	n/a	-	n.a.	-
93	FTLD-TDP type E	OND	F	51	-	0	n/a	-	n.a.	-
94	FTLD-FUS	OND	M	50	-	0	n/a	-	n.a.	-
95	ALS	OND	M	71	-	0	n/a	-	n.a.	-
96	Multiple sclerosis	OND	M	54	-	0	n/a	-	n.a.	-
97	FXTAS/NIID	OND	M	84	-	0	n/a	-	n.a.	-

98	Vascular dementia	OND	M	80	-	0	n/a	-	n.a.	-
99	Hydrocephalus adultorum	OND	F	70	-	0	n/a	-	n.a.	-

Abbreviations: AD, Alzheimer's disease; ALS, amyotrophic lateral sclerosis; ARTAG, age-related tau astrogliosis; CBD, corticobasal degeneration; CN, control; DLB, dementia with Lewy bodies; -FUS, frontotemporal lobar degeneration-fused in sarcoma; FTLD, FXTAS, Fragile X associated tremor/ataxie syndrome; GCI's, glial cytoplasmic inclusions; LB stage, Lewy body Braak stage; LPC stage, Lewy pathology consortium stage; MSA, multiple system atrophy; -c/p, cerebellar variant and predominant parkinsonism; NIID, neuronal intranuclear inclusion disease; ON, optic nerve; PART, primary age-related tauopathy; PD, Parkinson's disease; PDD, Parkinson's disease dementia; PSP, progressive nuclear palsy; -TDP, TAR DNA-binding protein 43; n/a, not applicable; n.a., not available; +, present; -, absent.
